# Transvenous Access for Emergent Thoracic and Thoracoabdominal Aortic Aneurysm Repair in Patients Without Femoral Access

**DOI:** 10.1177/15266028231197972

**Published:** 2023-09-09

**Authors:** Alessandro Grandi, Catharina Gronert, Giuseppe Panuccio, Fiona Rohlffs, Daour Yousef al Sarhan, Tilo Kölbel

**Affiliations:** 1 German Aortic Center Hamburg, Department of Vascular Medicine, University Heart Center, University Medical Center Hamburg-Eppendorf, Hamburg, Germany

**Keywords:** transvenous, transcaval, aneurysm, BEVAR, access, thoraco-abdominal, physician modified, PMEG, occlusion, stenosis

## Abstract

**Purpose:**

To describe the technique of transvenous access for emergent endovascular repair of thoracic and thoracoabdominal aneurysms exemplified with 2 cases.

**Technique:**

Transvenous access to the aorta is described as an alternative access method to deliver aortic endografts in emergency situations. A 68-year-old female patient with severely compromised iliac and subclavian artery access was treated for a ruptured extent V thoraco-abdominal aortic aneurysm with a t-Branch (Cook Medical, Bjaeverskov, Denmark) delivered through a transcaval access. To avoid severe aortocaval shunting a balloon-expandable covered stent was deployed through a carotid access due to severe bilateral subclavian ostial stenosis. A 71-year-old man with an acute type B aortic dissection and bilateral narrow long-segment stenting of the iliac arteries was treated with a physician-modified thoracic endovascular aortic repair using an arteriovenous fenestration created at the level of the common iliac artery. We describe the access creation by fenestration using a transseptal needle.

**Conclusion:**

Transvenous access for thoracic and thoraco-abdominal aortic aneurysm repair is safe and feasible in selected emergent cases.

**Clinical Impact:**

A transvenous approach may be helpful in selected patients when an endovascular repair needs to be performed but no arterial femoral access is available. This approach proved to be feasible even with large-bore introducer sheaths, taking its place in the armamentarium of the vascular surgeon for emergent complex endovascular aortic repairs.

## Introduction

Endovascular aortic repair has become the treatment of choice for thoracic aneurysms (thoracic endovascular aortic repair [TEVAR]) and is gaining popularity as first-line treatment for thoraco-abdominal aortic aneurysms (TAAA). An important feasibility criterion for endovascular repair is vascular access, which may preclude repairs in up to 20% of patients.^1^ Due to their large-bore profile, the delivery systems require suitable arterial access vessel diameters. The common femoral artery has been the traditional first choice due to its diameter superficial course with the femoral head allowing for safe compression.^2^ However, in patients with severe atherosclerotic disease, stenosis and occlusions of the iliofemoral axis are not uncommon and this may require alternative techniques or accesses to perform the procedures.^3^ Rogers et al^4^ recently described their experience in complex endovascular aortic repair in patients with unilateral occlusive disease as more challenging, but feasible with satisfactory outcomes, while Dias-Neto et al^5^ analyzed the use of iliofemoral conduits as a simultaneous or staged procedure and found no occurrence of inadvertent iliac artery disruption or conversion.

Since its first pre-clinical evaluation in 2013 by Halabi et al,^6^ transcaval access (TCA) has been increasingly utilized during transcatheter aortic valve repairs with good technical success rates. It has been proven to be safe and feasible for patients without arterial access^7^ or morbidly obese.^8^ Previous cases of TCA for TEVAR have been published,^9–11^ but no endovascular TAAA repair using TCA has been described to date.

We present 2 cases of transvenous access for the emergent endovascular repair of a thoracic and a TAAA treated in a national referral center for aortic disease with extensive experience in complex endovascular repairs.

## Technique

### Case 1

**Figure 1.**
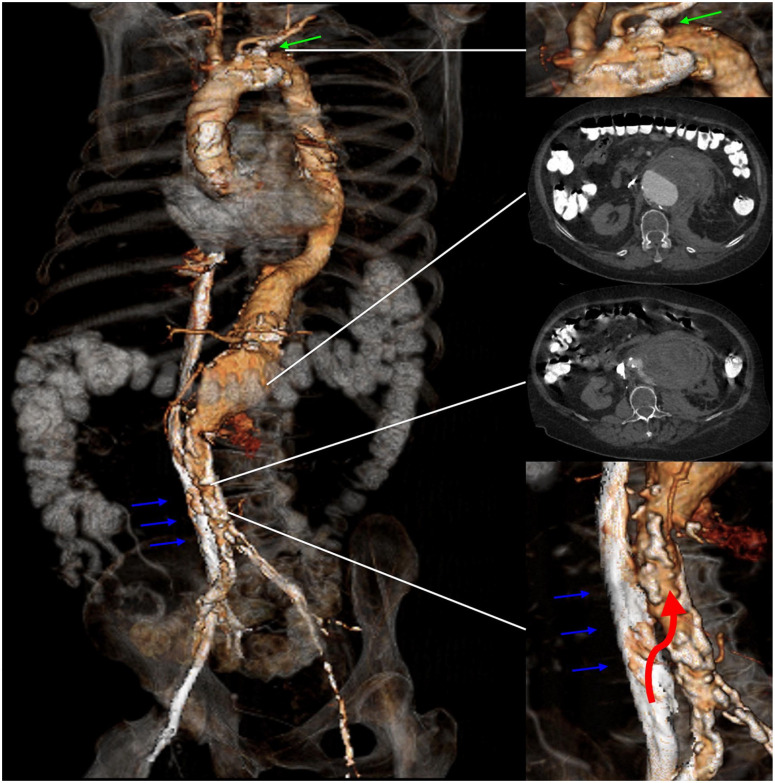
Three-dimensional rendering of the preoperative computed tomography scan with 2 axial views. Green arrow indicates the stenosis at the level of the left subclavian artery. Blue arrows indicate the inferior vena cava. Red arrow indicates the access point to the aorta from the inferior vena cava.

A 68-year-old woman with a history of severe chronic obstructive pulmonary disease, multiple sclerosis, pacemaker implantation, chronic kidney disease (CKD) with an atrophic left kidney and hypertension was transferred due to acute abdominal pain and hemoglobin drop (16–6.8 mg/dL) to our service. A computed tomography angiography (CTA) had revealed a ruptured type V TAAA. Furthermore, the CTA revealed a severely compromised iliofemoral axis with occlusion of the left iliac arteries and a long-segment high-grade stenosis of the right iliac artery with a luminal diameter of only 1 mm. Both subclavian arteries had severe ostial stenosis (Figure 1). Emergent endovascular repair appeared feasible using a t-Branch (Cook Medical, Bjaeverskov, Denmark) despite the lack of suitable iliofemoral access. A short infrarenal aortic segment appeared feasible for TCA.

### Procedure

**Figure 2.**
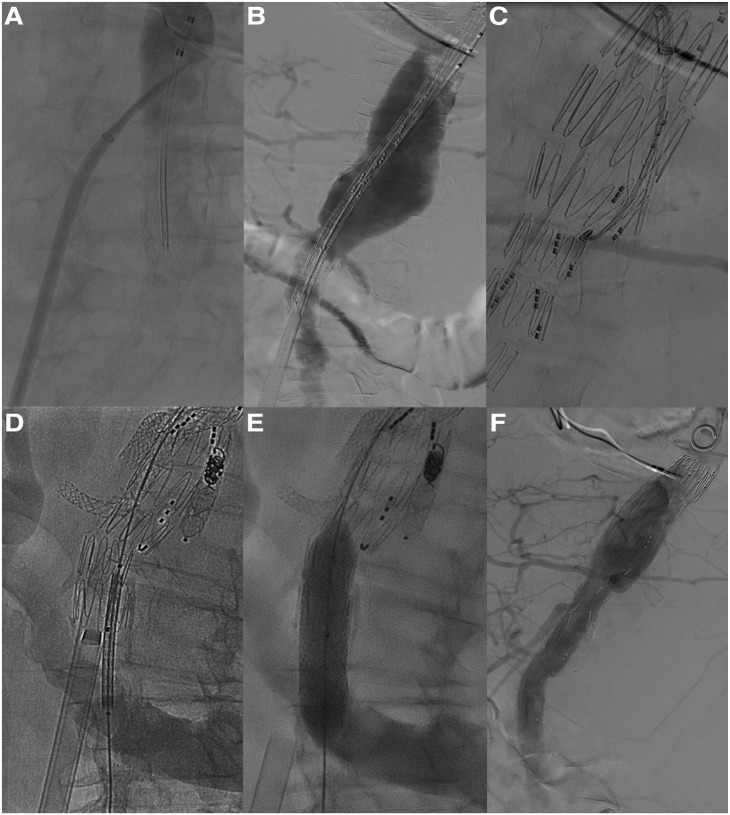
Intraoperative angiography depicting (A) angiographic check of the aorta after the transseptal needle puncture from the inferior vena cava, (B) introduction of the endograft in the aorta through the fenestration created from the inferior vena cava, (C) cannulation of the target vessels using a steerable sheath, (D and E) introduction and deployment of a covered stent to close the fenestration, (F) final angiographic check confirming correct exclusion of the aneurysm, patency of the target vessels and no arteriovenous flow at the fenestration site.

**Figure 3.**
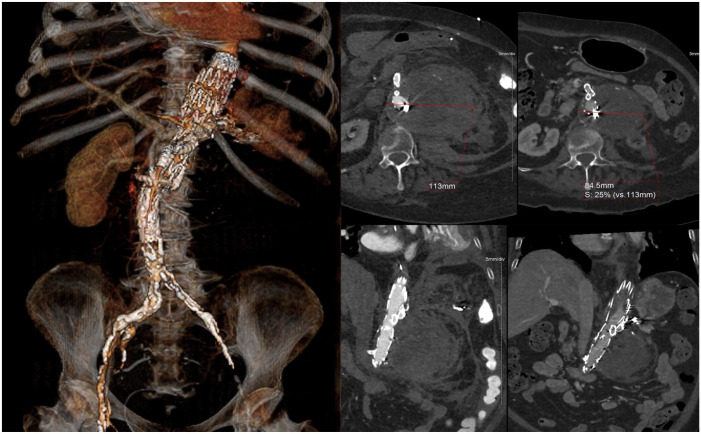
Three-dimensional rendering of the 6-month postoperative computed tomography scan and axial views showing shrinkage of the aneurysm sac. Red arrow indicates the access point to the common iliac artery from the common iliac vein.

Under general anesthesia, arterial access was obtained to the right common carotid artery (RCCA). A 5F sheath was inserted after systemic heparinization and a calibrated straight angiographic catheter was positioned in the distal descending thoracic aorta. The right common femoral vein was used for venous access with an 8F sheath (Cook Medical). A 63-cm long, 8F SL0 sheath dilator system (St Jude Medical, St Paul, Minnesota) was introduced over its guidewire into the inferior vena cava (IVC). The sheath was positioned against the IVC wall in proximity to the aneurysm. A Brockenbrough needle (Medtronic, Minneapolis, Minnesota) was coaxially introduced into the dilator. The aneurysm was then punctured with the sheath-dilator-needle system under fluoroscopic guidance using multiple projections. After successful puncture was confirmed by angiography, the dilator tip was forced into the aneurysm sac and the SL0 sheath was exchanged for an 8F 30 cm Flexor sheath (Cook Medical) over the SL0 guidewire to allow the use of a standard length (65 cm) catheter. The correct aortic puncture site was determined based on anatomical landmarks, CTA fusion with Vessel Navigator (Philips Healthcare, Best, The Netherlands) and multiple fluoroscopic projections. After predilatation using a 16F and 20F dilator, the t-Branch (Cook Medical) was introduced and deployed under digital subtraction angiography (DSA) and using preoperative CTA fusion (Vessel Navigator; Philips Healthcare). After deployment, the delivery system (22F inner diameter [ID]) was replaced by a 22F 25 cm Check-Flo sheath and a 14F 45 cm Check-Flo sheath (both Cook Medical) and a 10F 55 cm FuStar sheath (Lifetech Scientific Corp, Shenzhen, China) stabilized with a 0.014″ wire was used for transfemoral completion of the procedure bridging the celiac artery (CA), superior mesenteric artery (SMA), and right renal artery (RRA) in a standard fashion.^12,13^ The left renal artery branch was occluded after extension with a 6 mm 38 mm Advanta V12 (Atrium Medical Corporation, Merrimack, New Hampshire, USA) with an Amplatzer Vascular Plug II 10 mm (AGA Medical Corporation, Minneapolis, Minnesota) and a Nester coil 8 mm (Cook Medical).^14^ A 12F 80 cm Flexor sheath (Cook Medical) was placed from the antegrade RCCA access and a BeGraft Aortic 16×48 mm (Bentley InnoMed GmbH, Hechingen, Germany) was positioned at the level of the TCA in the infrarenal aorta. The BeGraft Aortic was deployed while the 22F Check-Flo sheath was retracted into the IVC. At completion angiography, patency of all 3 target vessels was confirmed with no type I/III endoleaks or relevant arteriovenous shunting to the IVC (Figure 2). The patient was extubated in the operating room and transferred to the intensive care unit where she stayed for 12 days due to pneumonia and need for non-invasive ventilation. The patient was discharged at home on postoperative day 15 without further complications (Figure 3).

### Case 2

**Figure 4.**
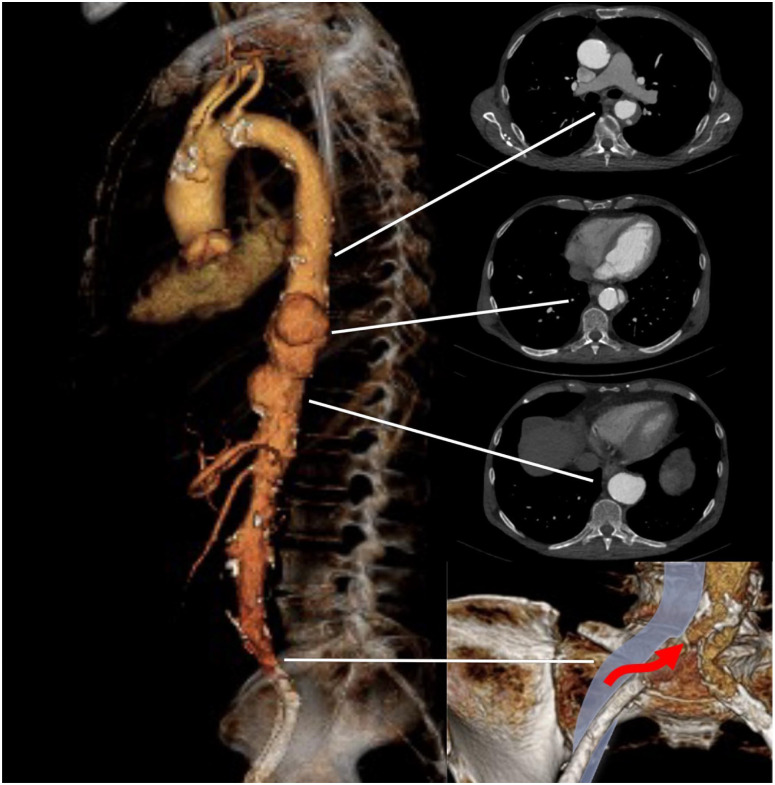
Three-dimensional rendering of the preoperative computed tomography scan with 3 axial views. Red arrow indicates the access point to the common iliac artery from the common iliac vein.

A 71-year-old man with a history of sarcoidosis (liver, kidneys, and lungs), metastasized colonic cancer, peripheral artery disease with bilateral iliac artery stenting, and dialysis-dependent CKD presented to the emergency department for acute thoracic pain. A CTA showed a type III thoraco-abdominal pseudoaneurysm from the mid descending thoracic aorta to the celiac trunk (CT). Both iliac arteries showed severe intrastent restenosis of the 6 mm long-segment self-expandable stents and both internal iliac arteries occluded (Figure 4). After a week of watchful waiting, the patient had a second episode of sudden thoracic pain and the CTA performed showed increased aortic diameters and a retrograde dissection of the TA to the left subclavian artery (LSA). Due to the marked progress, a decision was made to treat the patient urgently by a TEVAR and a physician-modified endograft (PMEG) with a distal scallop for the computed tomography (CT). Both iliac access sides were evaluated and deemed unsuitable for access with the 22F (ID) delivery system due to the small diameter (6 mm) self-expandable stents and the severe long-segment atherosclerotic external iliac arteries. The decision was made for the endografts to be delivered through a right iliofemoral transvenous access with preoperative cerebrospinal fluid drainage.

### Procedure

**Figure 5.**
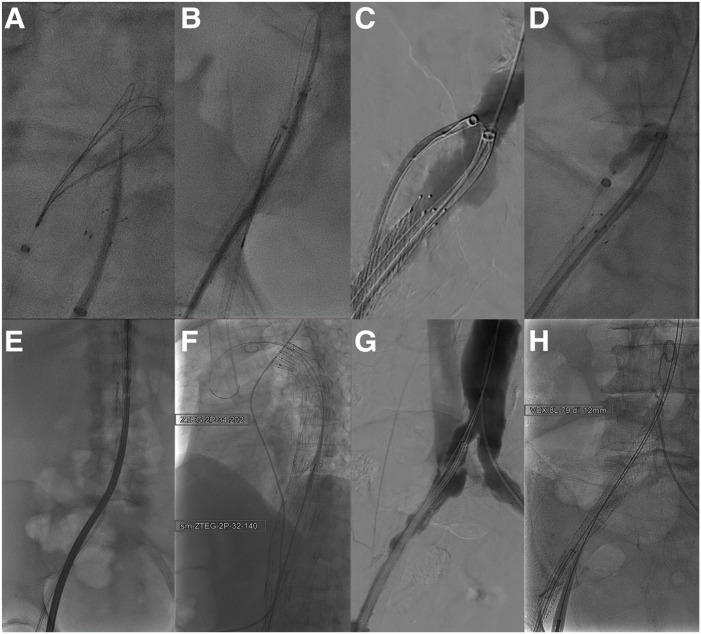
Intraoperative angiography depicting (A) the transseptal puncture from the common iliac artery to the common iliac vein, (B) snaring of the guidewire, (C) advancement of the venous introducer sheath at the fenestration level, (D) ballooning of the fenestration, (E) introduction of the endograft through the fenestration, (F) endografts after deployment, (G) angiographic check to see check the correct level of covered stent deployment, and (H) deployment of the covered stent to close the fenestration.

**Figure 6.**
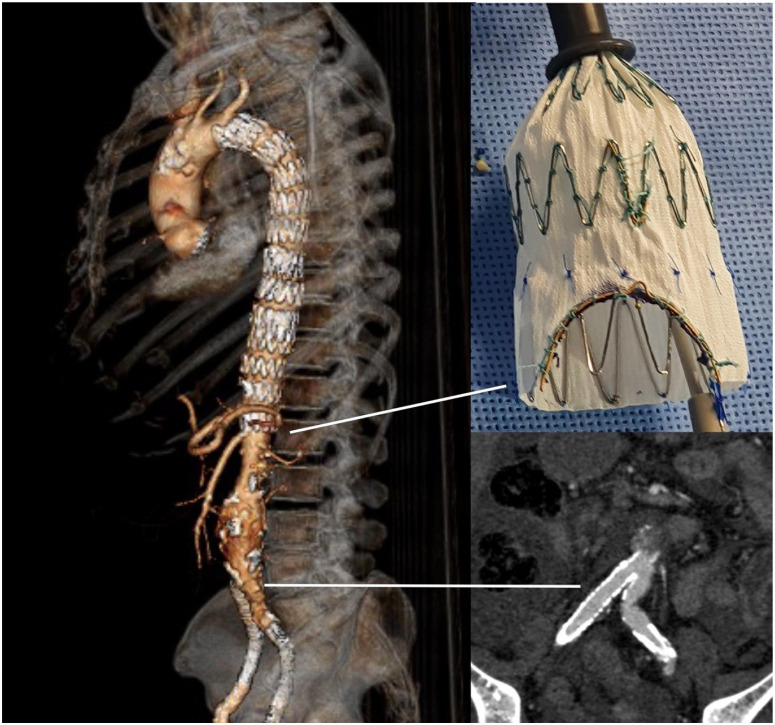
Three-dimensional rendering of the postoperative computed tomography scan with photograph of the scallop created in the distal end of the endograft for the celiac trunk and multiplanar reconstruction to show the patency of the covered stent implanted in the iliac artery with no sign of perfusion to the fenestration.

A back-table modification of a ZDEG-2P-32-140 (20F ID, Cook Medical) to create an 18×20 mm distal scallop was performed^15^ (Figure 5). Under general anesthesia, venous access was obtained at the right femoral vein and arterial access was obtained percutaneously at the right common femoral artery with a 7F introducer sheath. Subsequently, a 63-cm long, 8F SL0 sheath dilator system (St Jude Medical) was introduced over its guidewire on the arterial side and advanced at the level of the common iliac artery, carefully crossing the stenotic axis. The sheath was positioned against the iliac wall in proximity to common iliac vein. A Brockenbrough needle (Medtronic) was coaxially introduced into the dilator. Once in place, access to the common iliac vein was achieved through a puncture of both arterial and venous walls with the sheath-dilator-needle system under fluoroscopic guidance using multiple projections. After successful puncture was confirmed by angiography, a 0.014″ guidewire was passed through the fenestration and snared with a 5F Ensnare (Merit Medical System) from the venous side. The created fenestration was first dilated from the venous side with an 8×20 mm Advance angioplasty balloon (Cook Medical), followed by mechanical dilatation up to 20F. The PMEG, inserted from the venous side and advanced into the descending thoracic aorta through the fenestration created over an extra stiff guidewire, was deployed under fluoroscopy and preoperative CTA fusion (Vessel Navigator; Philips Healthcare) with the scallop correctly positioned at the level of the CT. A second endograft (ZDEG-2P-34-202, 20F ID, Cook Medical) was deployed proximally to the first, with the proximal markers at the level of the LSA. The overlap zone was ballooned with a Coda Balloon (Cook Medical). After removal of the devices from the venous side, an 8 mm VBX (WL Gore & Associates, Inc., Flagstaff, Arizona) was deployed and postdilated to 12 mm at the level of the fenestration in the common iliac artery to close it and avoid any possible arteriovenous shunting. Completion angiography and the postoperative CTA documented correct positioning of the endograft, patency of the LSA, CT, and common iliac artery. No arteriovenous fistula or extravasation of contrast was identified (Figure 6). The patient was extubated in the operating room and moved to the ward; he suffered from hepatitis and required ascites percutaneous aspiration and a urinary tract infection that required antibiotic treatment. After stabilization, the patient was discharged on postoperative day 12.

## Discussion

Access during endovascular aortic repair is of utmost importance to understand whether a patient is treatable or not. Here, 2 emergent cases in which no feasible arterial access routes were present. The use of a TCA has been thoroughly explored for transcatheter aortic valve implantation (TAVI) and endoleak treatment after endovascular aneurysm repair (EVAR).^16^ It presents a viable access route for large-bore devices with a high technical success rate and low perioperative complications.^17,18^

A recent systematic review on TCA for endoleak management by Nana et al^19^ included 8 papers, reporting on 128 procedures in 117 patients, and described high technical success, low mortality, and low rate of endoleak persistence after treatment. Few case reports have been published on the use of a TCA for TEVAR. While not the first-line access route, this approach should be considered in cases where occluded or very stenotic iliac arteries may preclude the repair. In the presented cases, due to the emergent nature of the pathology, the transvenous/transcaval approach was thought to be the fastest way to repair the aneurysms, as opposed to perform extra-anatomical conduits and proved safe even in complex endovascular repair.^5^

While other possible approaches were considered, none was considered viable in the presented cases. The “pave and crack” technique, consisting in the deployment and postdilatation of a covered stent graft to dilate as much as possible the iliac arteries, would not have been able to dilate the vessel enough to ensure the deployment of the endograft. The transapical access is an invasive surgical exposure, which would not have been possible due to the frailty of the treated patients, it is not considered a risk-free access. Intravascular lithotripsy could have been a valid alternative for an elective case, but due to the emergent nature of the described cases, the material was not available in stock.

When performing a TCA, the transseptal needle is inserted in the venous side and used to get access to the large aortic aneurysm sac. This is because going from a smaller caliber vessel (IVC) to a bigger caliber vessel (aorta) increases your chances of success. When applying the same concept to the iliac vein, the same result may not always be achieved. This may be due to the calcific status of the artery, as well as the smaller caliber that may hamper alignment of the needle with the vessel. In this situation, technical success may be achieved by inverting the sequence and going from artery to vein. The TCA can be performed in extent I/V TAAA, which may require iliac extension, while transvenous iliac access should be chosen in extent II/III/IV, which usually requires iliac extensions.

At the end of the aortic repair, the arteriovenous fistula that was created distally to the distal landing zone needs to be closed in order to avoid right heart failure. In the present cases, due to the emergent nature of the repairs, a covered stent was placed at the level of the fenestration on the arterial side achieving complete exclusion both at completion angiography and follow-up CTA.

Finally, anatomical suitability for transvenous/caval access should be carefully evaluated on preoperative CTA.^20^ This is done by identifying aortic segments with no calcium plaques targets on the right wall of the aorta, no interposed bystander structures, and distant from important vascular branches.

Transcaval access in emergent procedures may also have some limitations. As stated, patient suitability needs to be carefully examined as the presence of calcium plaques in the aorta may preclude TCA use, as well as a large distance between the aorta and the IVC may increase the odds of damaging interposed structures with the needle while creating the fenestration. Therefore, careful patient selection is mandatory when opting for this approach.

## Conclusion

Transvenous and transcaval approaches for thoracic and TAAA repair are safe and feasible in emergent cases where femoral accesses are not available. Larger experiences should be gathered to understand its possible implications in elective cases, building on the literature available for transcatheter aortic valve repairs.
